# Simulating transmission of ESKAPE pathogens plus *C. difficile* in relevant clinical scenarios

**DOI:** 10.1186/s12879-020-05121-4

**Published:** 2020-06-12

**Authors:** Katharina L. Weber, Danielle S. LeSassier, Anthony D. Kappell, Kathleen Q. Schulte, Nicole Westfall, Nicolette C. Albright, Gene D. Godbold, Veena Palsikar, Carlos A. Acevedo, Krista L. Ternus, F. Curtis Hewitt

**Affiliations:** Signature Science, LLC, Austin, TX USA

**Keywords:** Healthcare-acquired infections, Pathogen transmission, Epidemiology, Synthetic skin, ESKAPE pathogens, *C. difficile*, Decontamination

## Abstract

**Background:**

The prevalence of healthcare-acquired infections (HAI) and rising levels of antimicrobial resistance places significant economic and public health burdens on modern healthcare systems. A group of highly drug resistant pathogens known as the ESKAPE pathogens, along with *C. difficile*, are the leading causes of HAIs. Interactions between patients, healthcare workers, and environmental conditions impact disease transmission. Studying pathogen transfer under varying contact scenarios in a controlled manner is critical for understanding transmission and disinfectant strategies. In lieu of human subject research, this method has the potential to contribute to modeling the routes of pathogen transmission in healthcare settings.

**Methods:**

To overcome these challenges, we have developed a method that utilizes a synthetic skin surrogate to model both direct (skin-to-skin) and indirect (skin-to fomite-to skin) pathogen transfer between infected patients and healthy healthcare workers. This surrogate material includes a background microbiome community simulating typical human skin flora to more accurately mimic the effects of natural flora during transmission events.

**Results:**

We demonstrate the ability to modulate individual bacterial concentrations within this microbial community to mimic bacterial concentrations previously reported on the hands of human subjects. We also explore the effect of various decontamination approaches on pathogen transfer between human subjects, such as the use of handwashing or surface disinfectants. Using this method, we identify a potential outlier, *S. aureus,* that may persist and retain viability in specific transfer conditions better than the overall microbial community during decontamination events.

**Conclusions:**

Our work describes the development of an in vitro method that uses a synthetic skin surrogate with a defined background microbiota to simulate skin-to-skin and skin-to fomite-to skin contact scenarios. These results illustrate the value of simulating a holistic microbial community for transfer studies by elucidating differences in different pathogen transmission rates and resistance to common decontamination practices. We believe this method will contribute to improvements in pathogen transmission modeling in healthcare settings and increase our ability to assess the risk associated with HAIs, although additional research is required to establish the degree of correlation of pathogen transmission by skin or synthetic alternatives.

## Background

Healthcare-acquired infections (HAIs) and increasing levels of antimicrobial resistance, particularly among HAI-associated pathogens, represent a significant source of morbidity and mortality cost in the US healthcare system [1]. Understanding the dynamics of pathogen transmission and transfer between patients, healthcare workers, and surrounding environmental surfaces is critical to evaluate potential intervention methods, identify possible environmental HAI reservoirs or fomites, and to delineate pathogen-specific transmission behaviors [2–5]. Ethical restrictions on human subject research complicates experimental analysis of human-to-human pathogen transfer. Retrospective studies on HAI outbreaks do not provide experimentally changeable parameters. Thus, there is a need for in vitro methods to study these types of transmission scenarios.

The ESKAPE pathogens (*Enterococcus faecium*, *Staphylococcus aureus*, *Klebsiella pneumoniae*, *Acinetobacter baumannii*, *Pseudomonas aeruginosa*, and *Enterobacter* species) [6] are a group of Gram-positive and Gram-negative bacteria characterized by drug-resistance [6, 7]. Taken together with *Clostridioides difficile*, which is often caused by antibiotic-induced disruption of the native gut microbiota [8], these pathogens (here termed ESKAPE+C) collectively are the leading cause of nosocomial infections [6, 7]. These pathogens employ diverse mechanisms of drug resistance, such as drug inactivation, active site modification, efflux pumps, and biofilm formation, leading to multidrug-resistant (MDR) strains and a need for novel antibiotics [6, 7, 9, 10]. As a result of their clinical impact, the Centers for Disease Control and Prevention (CDC), with the Food and Drug Administration (FDA), maintains the Antibiotic Resistance (AR) Isolate Bank which maintains and curates multiple, well-characterized strains of ESKAPE pathogens and isolate panels, a resource that was utilized for this effort [11].

Previous studies have investigated surface transfer of individual ESKAPE+C pathogens, focusing on outcomes of possible transmission events. Arinder et al. utilized VITRO-SKIN to show transfer rates of *S. aureus* to and from various fomites, but the study did not include relevant background microbiota [12]. Dyer et al. investigated *C. difficile* transmission from hospital fomites by directly applying spores to the fomite surface, which may not be representative for most transmission scenarios [13]. These studies take diverse approaches to investigating microbial transfer, making comparison of findings and transmission rates challenging. A standardized approach that is broadly applicable to relevant HAI pathogens would be beneficial in modeling pathogen transmission in healthcare-like scenarios.

In the current study, an in vitro method to enable investigation of both direct (skin-to-skin) and indirect (skin-to fomite-to skin) transmission scenarios of ESKAPE+C pathogens is presented. This method utilizes a commercially available synthetic skin surrogate, VITRO SKIN® N-19, to mimic human skin and allow more accurate assessment of bacteria transmission behavior under various direct and indirect scenarios. We describe the inclusion of a set of background organisms commonly found on human skin, recapitulating the native microbiota present during such transmission events. Further, washing approaches for both VITRO-SKIN and relevant porous and nonporous surfaces (i.e., stainless steel, nitrile, and cotton) are presented. Using this method, ESKAPE+C pathogens were found to transfer at different rates and the inclusion of washing methods for indirect, but not direct, scenarios generally reduced the transfer burden. The described approach is highly customizable, allowing researchers to investigate a specific surface, disinfectant methods, or pathogen(s) as desired.

## Results

### Simulating direct and indirect contact scenarios

**Fig. 1:**
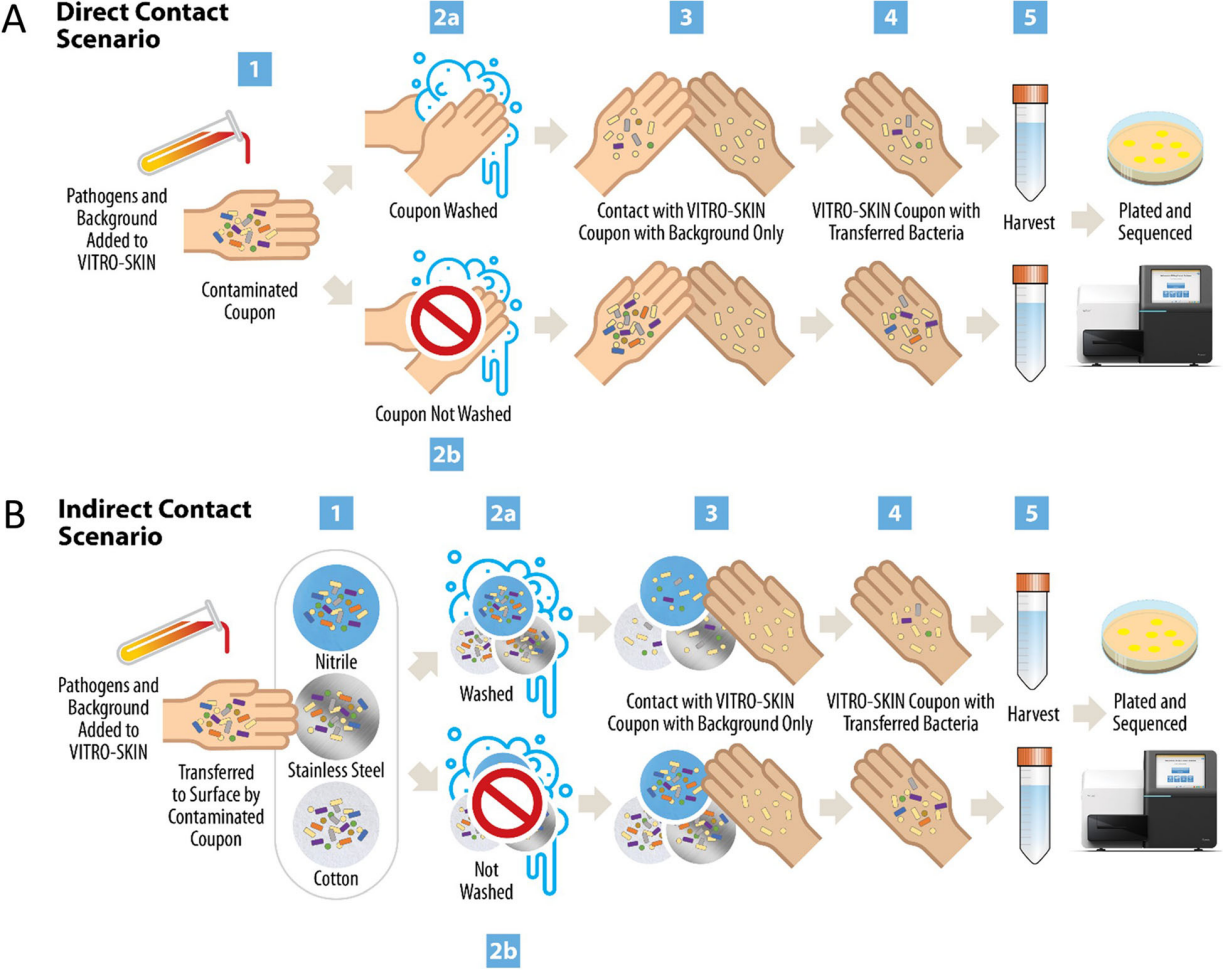
Approach for the Assessment of ESKAPE+C Pathogen Transmission for Direct and Indirect Transmission Events. **a** For the direct contact scenario, the primary VITRO-SKIN coupon is inoculated with a mix of pathogen and background bacteria, representing a contaminated patient hand (step 1). The inoculated VITRO-SKIN is either washed (step 2a) or not washed (step 2b) and then touched to a new, secondary VITRO-SKIN with only background microorganisms (step 3), simulating the touch transfer of bacteria from a sick patient to a clean healthcare worker. The secondary VITRO-SKIN with any transferred bacteria (step 4), representing the contaminated healthcare worker, is then harvested for downstream analysis (step 5). **b** For the indirect contact scenario, the primary VITRO-SKIN coupon is inoculated with a mix of pathogen and background bacteria, representing a contaminated patient hand. The inoculated VITRO-SKIN is touched to a surface (nitrile, stainless steel, or cotton), simulating bacterial transfer to the fomite (step 1). The fomite then either undergoes a surface-appropriate wash (step 2a) or does not (2b). A new, secondary VITRO-SKIN with only background microorganisms, representing a clean health worker hand, touches the fomite (step 3) where bacteria on the surface may transfer to the secondary VITRO-SKIN (step 4). The secondary VITRO-SKIN with any transferred bacteria is then harvested for downstream analysis (step 5)

Bacterial transmission can occur through either “direct” skin-to-skin contact, such as between an infected patient and a healthcare worker, or an “indirect” skin-to fomite-to skin transfer. We constructed a workflow to mimic these contact scenarios in order to investigate direct and indirect ESKAPE+C pathogen transfer utilizing VITRO-SKIN, a synthetic skin material (Fig. 1). As pathogen transfer does not occur in isolation, a set of “background” bacteria were included to represent the native skin microbiota that could be present on human hands. Table 1 lists the ESKAPE+C and background organisms used for this work. To simulate direct contact, a primary VITRO-SKIN coupon (i.e., infected patient) was briefly touched to a secondary VITRO-SKIN coupon containing only background organisms (i.e., healthy healthcare worker with a healthy skin flora) with or without a simulated handwashing step (Fig. 1a). For indirect contact, the primary VITRO-SKIN coupon was touched to an intermediate fomite (i.e., cotton, nitrile glove, or stainless steel). The surface was either washed or not washed before contact with the secondary VITRO-SKIN coupon (Fig. 1b). In both scenarios, viable pathogens were then recovered from the secondary coupon and enumerated.

**Table 1:** Bacteria used in this study

Background Microorganisms	Pathogenic Microorganisms
*Brevibacterium linens* ATCC 9172	*Acinetobacter baummannii* AR Bank 275
*Corynebacterium matruchotii* ATCC 14266	*Enterococcus faecium* AR Bank 579
*Cutibacterium acnes* ATCC 11827	*Klebsiella pneumoniae* AR Bank 139
*Escherichia coli* ATCC 9637	*Enterobacter aerogenes* AR Bank 161
*Lactobacillus gasseri* ATCC 33323	*Staphylococcus aureus* AR Bank 219
*Micrococcus luteus* ATCC 4698	*Clostridioides difficile* (endospores) ATCC 43598
*Staphylococcus epidermidis* ATCC 12228	*Enterobacter cloacae* AR Bank 365
*Streptococcus pyogenes* ATCC 49399	*Pseudomonas aeruginosa* AR Bank 230

### Evaluation of bacterial loading on simulated skin

**Fig. 2:**
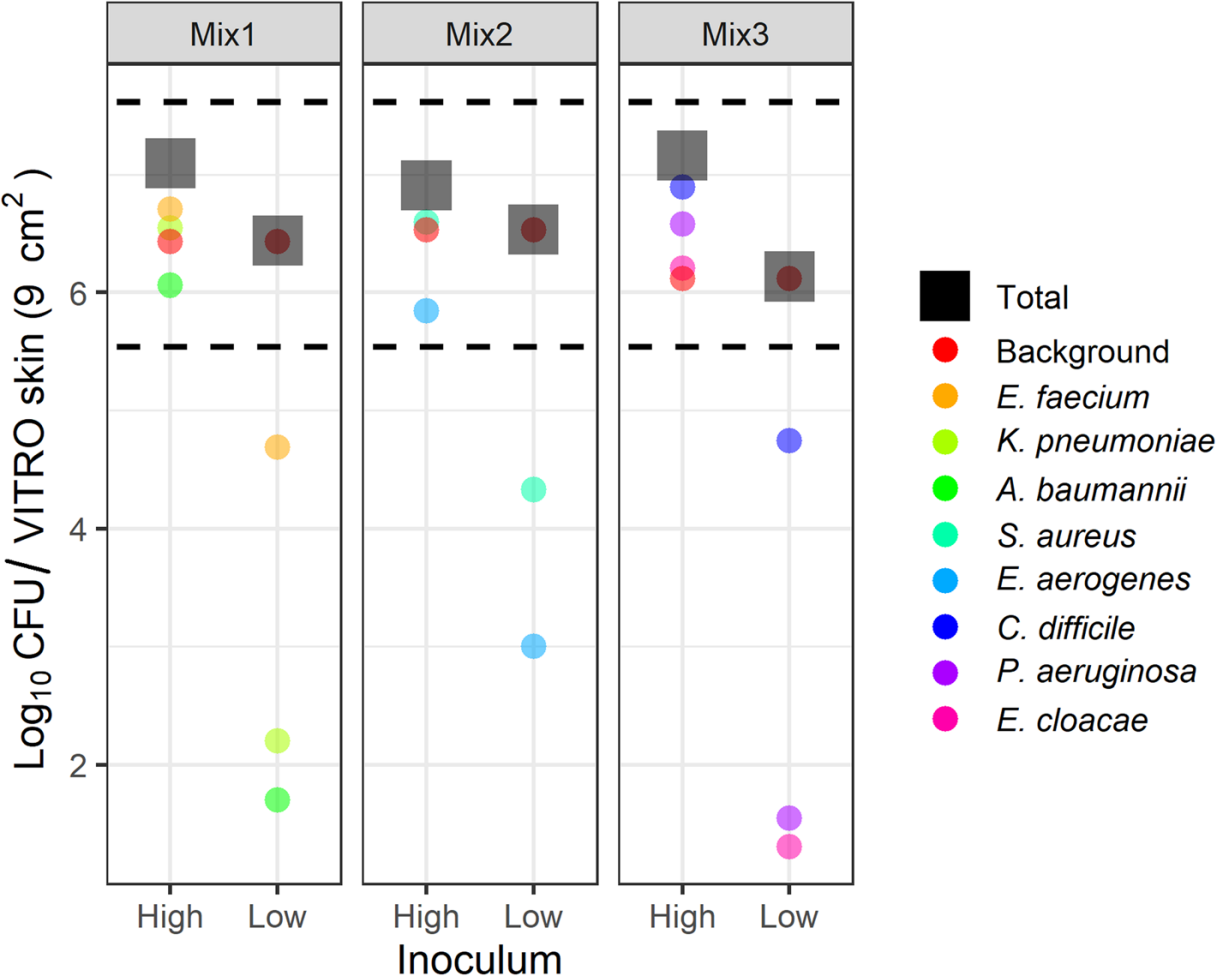
Representative Bacterial Loadings on the Initial VITRO-SKIN Coupon. The ESKAPE+C pathogens were grouped into three separate mixes to facilitate culture-based screening at either high or low levels relative to a consistent background microbial community. Total loadings (black squares) for both the high and low inoculum levels fell within equivalent reported ranges (denoted by black dotted lines) of bacterial loads on the hands of healthcare workers (between 3.9 × 10^4^ and 4.6 × 10^6^ CFU / 9 cm^2^) [14]

Total bacterial loadings on the initial simulated skin coupon were quantified to ensure concordance with previously reported bacterial concentrations on the human hand. Published reports describing typical total microbial content on human hands in healthcare settings generally range between 3.9 × 10^4^ and 4.6 × 10^6^ colony forming units (CFU)/cm^2^ [14]. To better simulate a range of pathogen transmission scenarios, different amounts of pathogen were added to a consistent background microbiome community. The pathogens were grouped into three mixes to better enable analysis by plating on selective media (Table 1). The “high” concentration samples contained approximately equivalent amounts of each microbial component (10^5^ CFU/cm^2^ or 10^6^ CFU per 9 cm^2^ coupon). Pathogen amounts in the “low” concentration samples were 100–1000 fold less abundant in comparison. The amount of each pathogen, the background community, and the total amount of bacteria loaded onto the simulated skin coupon is shown in Fig. 2.

### Pathogen transmission by direct contact

**Fig. 3:**
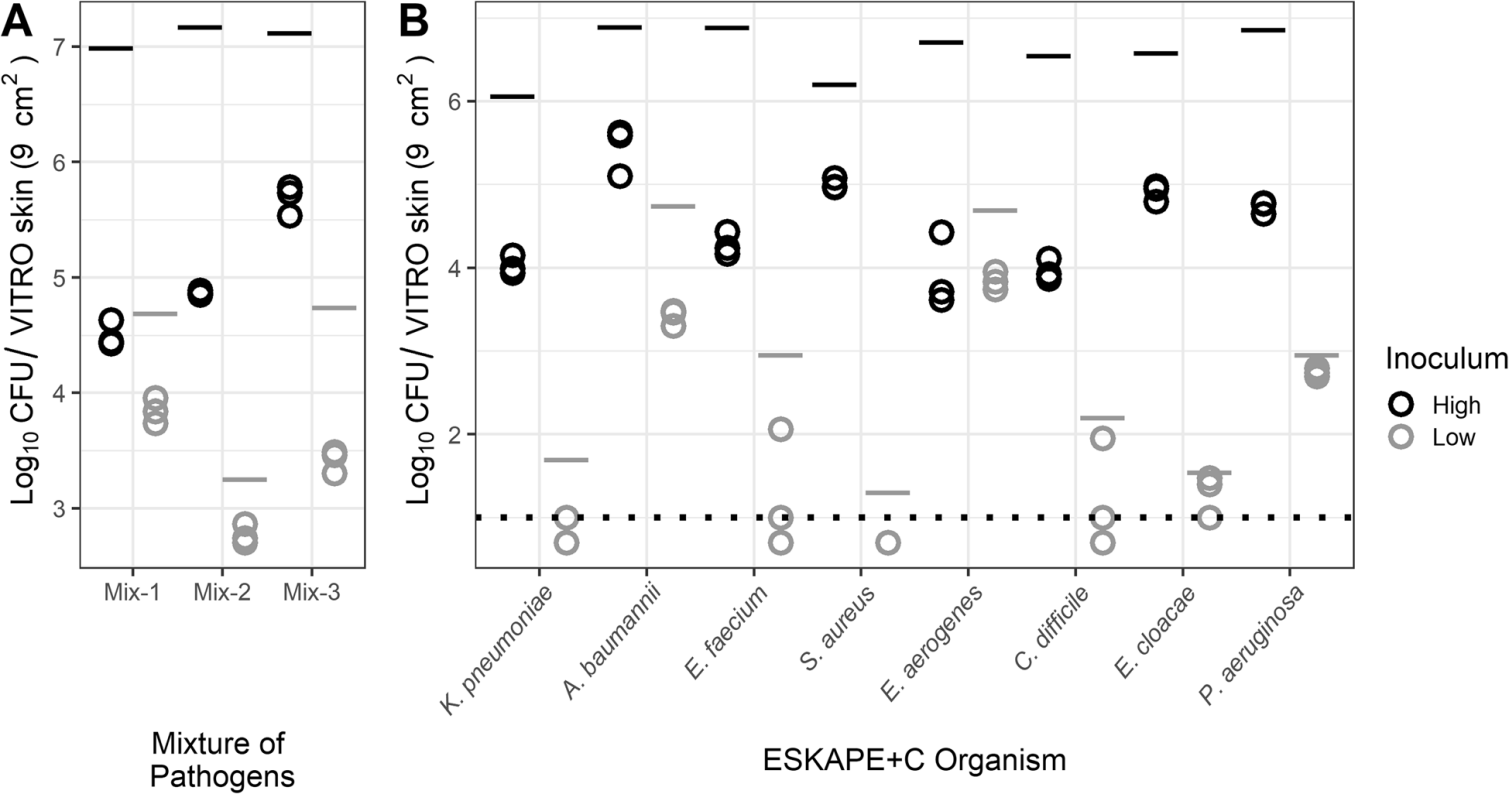
Direct transfer of ESKAPE+C Organisms performed in mixture with high and low initial inoculum. **a** Total amount of pathogen recovered from secondary VITRO-SKIN coupon following direct transfer. The CFU transferred (Log_10_) within mixtures are represented by the sum of the CFU for each pathogen averaged across three replicates. **b** Total amount of individual ESKAPE+C organisms within the microbial community mixture recovered from the secondary VITRO-SKIN coupon following direct transfer. Each pathogen is represented by the mean total CFU for each pathogen across three replicates. Black or gray lines above each point indicate initial pathogen spike levels on the primary VITRO-SKIN coupon. Dotted-line represents the limit of detection. Points below the dotted line result from averaging between replicates when one replicate had zero CFU

The overall level of ESKAPE+C pathogen transfer from the primary VITRO-SKIN coupon to a secondary VITRO-SKIN coupon following direct contact was established by measuring the total CFU recovery from the secondary coupon. Figure 3 shows the total CFU (log_10_) of each pathogen following transfer for each bacterial mix (3A) or individual pathogen (3B). Each individual replicate is plotted (*n* = 3). The total inoculum on the initial coupon is indicated for comparison.

*A. baumannii*, *E. aerogenes*, *E. faecium*, *K. pneumoniae*, *P. aeruginosa*, and *S. aureus* had significantly greater direct transfer with smaller log differences at lower inoculum ranging from 0.213 to 1.70 compared to higher inoculum with log differences ranging from 1.67 to 2.79 (*p* < 0.034). *C. difficile* (*p* = 0.999) and *E. cloacae* (*p* = 0.536) had no significant differences in direct transfer rates between the high and low inoculum used in this study. While the high inoculum samples generally transferred a greater number of total CFU in comparison with the lower inoculum samples (with the novel exception for *E. faecium*), higher pathogen concentrations led to lower overall pathogen transfer rates from the primary to the secondary coupon.

### Pathogen transmission by indirect contact

**Fig. 4:**
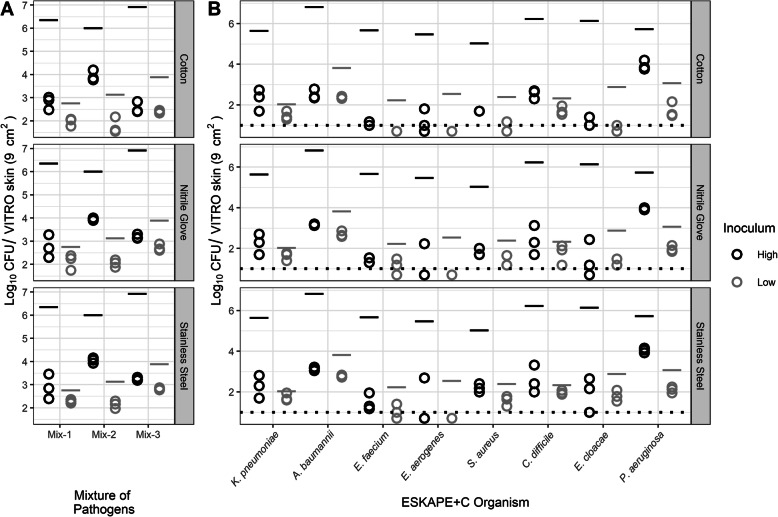
Indirect transfer of ESKAPE+C Organisms performed in mixture with high and low initial inoculum. **a** Total amount of pathogen recovered from secondary VITRO-SKIN coupon following indirect transfer across cotton, nitrile, and stainless steel surfaces. The CFU transferred (Log_10_) within mixtures is represented by the sum of the CFU for each pathogen averaged across three replicates. **b** Total amount of individual ESKAPE+C organisms within the microbial community mixture recovered from the secondary VITRO-SKIN coupon following indirect transfer across the three surface types. Each pathogen is represented by the mean total CFU for each pathogen across three replicates. Black or gray lines above each point indicate initial pathogen spike levels on the primary VITRO-SKIN coupon. Dotted-line represents the limit of detection. Points below the dotted line result from averaging between replicates when one replicate had zero CFU

Similar to the direct transfer testing described above, total CFU counts were measured for each mix or individual ESKAPE+C pathogen recovered from the secondary VITRO-SKIN coupon following indirect transfer. Indirect transfer involved transmission to a fomite (cotton, nitrile, or stainless steel) from the initial VITRO-SKIN coupon followed by transmission from the fomite to the secondary VITRO-SKIN surface. As with the direct transfer data, total CFUs were plotted for both high and low pathogen innocula (Fig. 4). Initial pathogen inoculum levels are indicated by lines for estimation of overall transfer rates across each fomite type.

Overall, indirect transmission led to significantly lower transmission rates than direct transfer (four-way ANOVA including the factor for type of transfer: F_(1,160)_ = 17.4684, *p* < 0.001) with CFU counts generally one to three orders of magnitude for high concentration innocula. A three-way analysis of variance (ANOVA) was conducted on the influence of inoculum, organism, and fomite type on the change in log concentration during indirect transfer of different mixtures of pathogen from VITRO-SKIN to a fomite to a VITRO-SKIN. The main effects inoculum (F_(1,96)_ = 1426.5, *p* < 0.001), organism (F_(7,96)_ = 39.4, *p* < 0.001), fomite (F_(2,96)_ = 10.4, *p* < 0.001), and the interaction term of inoculum and organism (F_(7,96)_ = 21.0, *p* < 0.001) were statistically significant. Post-hoc Tukey honest significant difference test indicated significant differences between indirect transfer with cotton fomite compared to nitrile gloves (*p* = 0.023) and stainless steel *(p* < 0.001), while there was no significant difference in transfer between stainless steel and nitrile gloves (*p* < 0.160). Similar to direct transfer of pathogens, there was no significant difference in log differences between high and low inoculum of *C. difficile* (*p* = 1.00) and *E. cloacae* (*p* = 0.536). The other pathogens, *A. baumannii, E. aerogenes, E. faecium, K. pneumoniae, P. aeruginosa*, and *S. aureus* had significantly lower differences in log CFU at lower inoculum, ranging from 0.43 to 1.60, than higher inoculum, ranging from 1.76 to 4.6, indicating greater relative abundance transfer (*p* < 0.005). The three-way interaction term of all main effects (F_(14,96)_ = 0.203, *p* = 0.999) and interaction terms including fomite with inoculum (F_(2,96)_ = 0.389, *p* = 0.679) or organism (F_(14,96)_ = 0.888, *p* = 0.574) were not statistically significant. *S. aureus* represented the exception in the indirect transfer data, transferring at a higher rate across all three surfaces than the other ESKAPE+C pathogens.

### Effect of simulated hand washing for direct contact scenarios

**Fig. 5:**
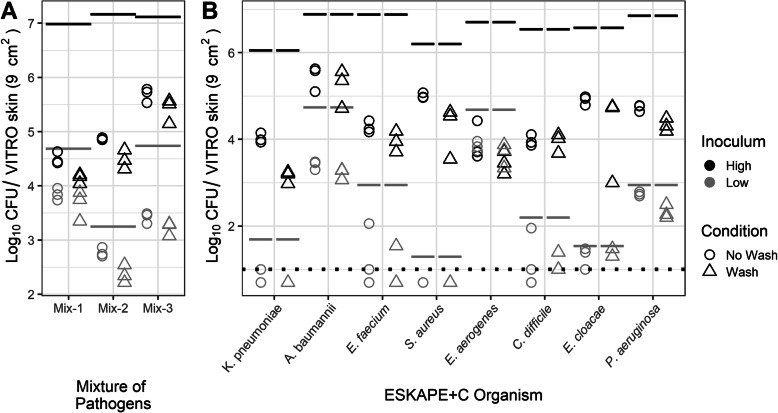
Effect of simulated hand washing on direct skin-to-skin transfer. **a** Total amount of pathogen recovered from secondary VITRO-SKIN coupon following direct transfer in the presence or absence of a simulated hand washing event. The CFU transferred (Log10) within mixtures are represented by the sum of the CFU for each pathogen averaged across three replicates. **b** Total amount of individual ESKAPE+C organisms within the microbial community mixture recovered from the secondary VITRO-SKIN coupon following direct transfer in the presence or absence of a simulated hand washing event. Each pathogen is represented by the mean total CFU for each pathogen across three replicates. Black or gray lines above each point indicate initial pathogen spike levels on the primary VITRO-SKIN coupon. Dotted-line represents the limit of detection. Points below the dotted line result from averaging between replicates when one replicate had zero CFU

The artificial skin method described herein provides opportunities to evaluate many facets of skin-to-skin pathogen transmission, including the effectiveness of hand washing or fomite decontamination methods. The effect of handwashing on direct transfer rates was simulated by manual scrubbing and immersion into a series of tubes containing phosphate buffered saline (PBS) to approximate rinsing. Overall, the effect of simulated hand washing was minimal, with washed samples generally demonstrating less than 10-fold reduction in transferred pathogen compared to non-washed samples (Fig. 5). A three-way ANOVA was performed on the influence of inoculum, organism, and washing on the change in log change during direct transfer of mixtures of pathogen between VITRO-SKIN. The main effects inoculum (F_(1,64)_ = 410.5, *p* < 0.001), organism (F_(7,64)_ = 17.2, *p* < 0.001), and washing (F_(1,64)_ = 17.5, *p* < 0.001), and the interaction terms with inoculum and organisms (F_(7,64)_ = 10.57, *p* < 0.001) or washing (F_(1,64)_ = 7.61, *p* = 0.008) were statistically significant. Post-hoc Tukey honest significant difference test indicated a significantly greater log difference, less transfer, after washing compared to unwashed scenarios (*p* < 0.001). Post-hoc testing also indicated that washing the high inoculum samples significantly increased log difference and led to less transfer, compared to not washing (*p* < 0.001). There were no significant differences at low inoculum between washing and not washing (*p* = 0.748). The three-way interaction term of all main effects (F_(7,64)_ = 0.821, *p* = 0.573) and the interaction term of washing and organisms (F_(7,64)_ = 0.706, *p* = 0.667) were not statistically significant.

### Effect of simulated fomite decontamination for indirect contact scenarios

**Fig. 6:**
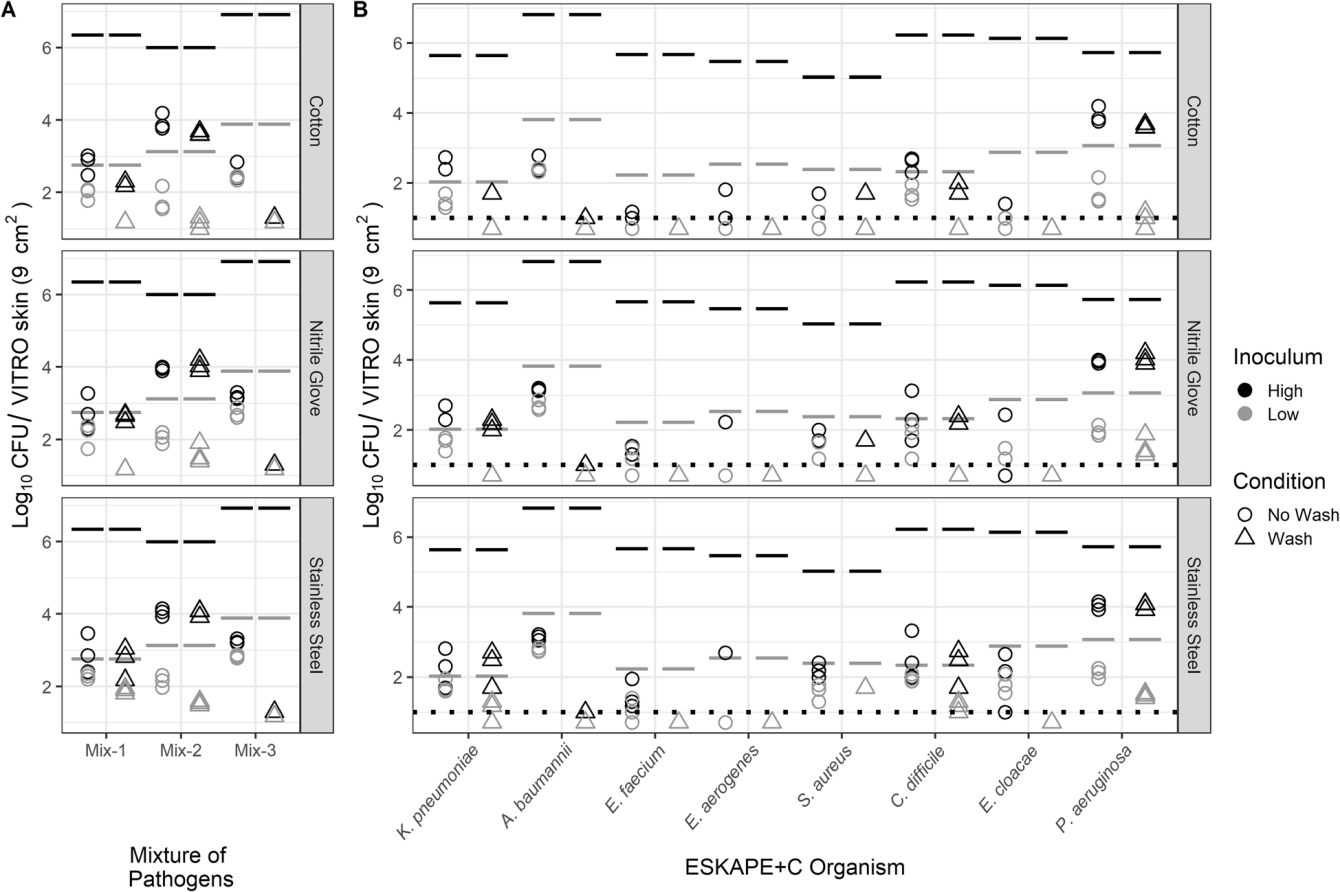
Effect of simulated fomite decontamination on indirect pathogen transfer. **a** Total amount of pathogen recovered from secondary VITRO-SKIN coupon following indirect transfer across cotton, nitrile, and stainless steel surfaces in the presence or absence of a simulated decontamination or washing event. The CFU transferred (Log_10_) within mixtures is represented by the sum of the CFU for each pathogen averaged across three replicates. **b** Total amount of individual ESKAPE+C organisms within the microbial community mixture recovered from the secondary VITRO-SKIN coupon following indirect transfer across the three surface types with or without simulated decontamination or washing. Each pathogen is represented by the mean total CFU for each pathogen across three replicates. Black or gray lines above each point indicate initial pathogen spike levels on the primary VITRO-SKIN coupon. Dotted-line represents the limit of detection. Points below the dotted line result from averaging between replicates when one replicate had zero CFU

The effect of fomite decontamination was also investigated (Fig. 6). Cotton samples were agitated in a soap solution to simulate laundering, while nitrile and stainless steel surfaces were swabbed with a disinfectant wipe. Cotton surfaces that were washed experienced a ≥ 6 log CFU reduction in bacterial abundance compared to the unwashed samples for the high spike-in. A four-way ANOVA was performed on the influence of inoculum, organism, fomite type, and washing on the differences in log change during indirect transfer of mixtures of pathogens from VITRO-SKIN to fomite to VITRO-SKIN. The main effects inoculum (F_(1,192)_ = 4915.73, *p* < 0.001), organism (F_(7,192)_ = 192.65, *p* < 0.001), fomite type (F_(2,192)_ = 25.05, *p* < 0.001), and washing (F_(1,192)_ = 331.67, *p* < 0.001) were statistically significant. Additionally the interaction terms of inoculum and washing (F_(7,192)_ = 88.67, *p* < 0.001), organism and washing (F_(7,192)_ = 30.47, *p* < 0.001), the three-way interaction term inoculum, organism, and washing (F_(7,192)_ = 5.21, *p* < 0.001), and three-way interaction term organism, fomite type, and washing(F_(14,192)_ = 2.04, *p* = 0.017) were also statistically significant. At low inoculum, the pathogens *A. baumannii, C. difficile, K. pneumoniae, P. aeruginosa*, and *S. aureus* had less indirect transfer and greater log change after washing (*p* < 0.013). *C. difficile, E. cloacae, E. aerogenes*, and *P. aeruginosa* at high inoculum had less indirect transfer after washing (*p* < 0.04). The indirect transfer of *E. faecium* at high and low inoculum was not statistically significantly different (p > 0.984) after washing. Washing was most effective against the indirect transfer of *C. difficile* from all three surfaces examined (*p* < 0.001). Washing also significantly reduced the indirect transfer of *K. pneumoniae* on cotton surfaces (*p* < 0.001) and *P. aeruginosa* on stainless steel (*p* < 0.001). Similar to direct and indirect transfer without washing, the pathogens had significantly smaller log changes and higher overall relative transfer rates with low inoculum compared to high inoculum (*p* < 0.001) when washed, with the exception of *S. aureus* which demonstrated no statistically significant difference (*p* = 1.00).

### Effectiveness of decontamination for the synthetic skin model

To assess the overall effectiveness of the decontamination methods utilized in this study, the percent of total pathogen transferred from the initial to the secondary coupon was calculated. All raw data used for these calculations, including total bacterial loadings and total pathogen transferred for each replicate are included in Supplemental Table 1. Table S1 includes the calculated transfer rate for each pathogen and each replicate. These transfer rate values were averaged for each pathogen at each condition (*n* = 3) and plotted. Regression analysis was performed to compare percent transfer between washed and unwashed samples. In this case, a slope of y = 1 would suggest no effect of washing on pathogen transfer, while a slope of y = 0 would demonstrate complete pathogen removal or decontamination.

**Fig. 7:**
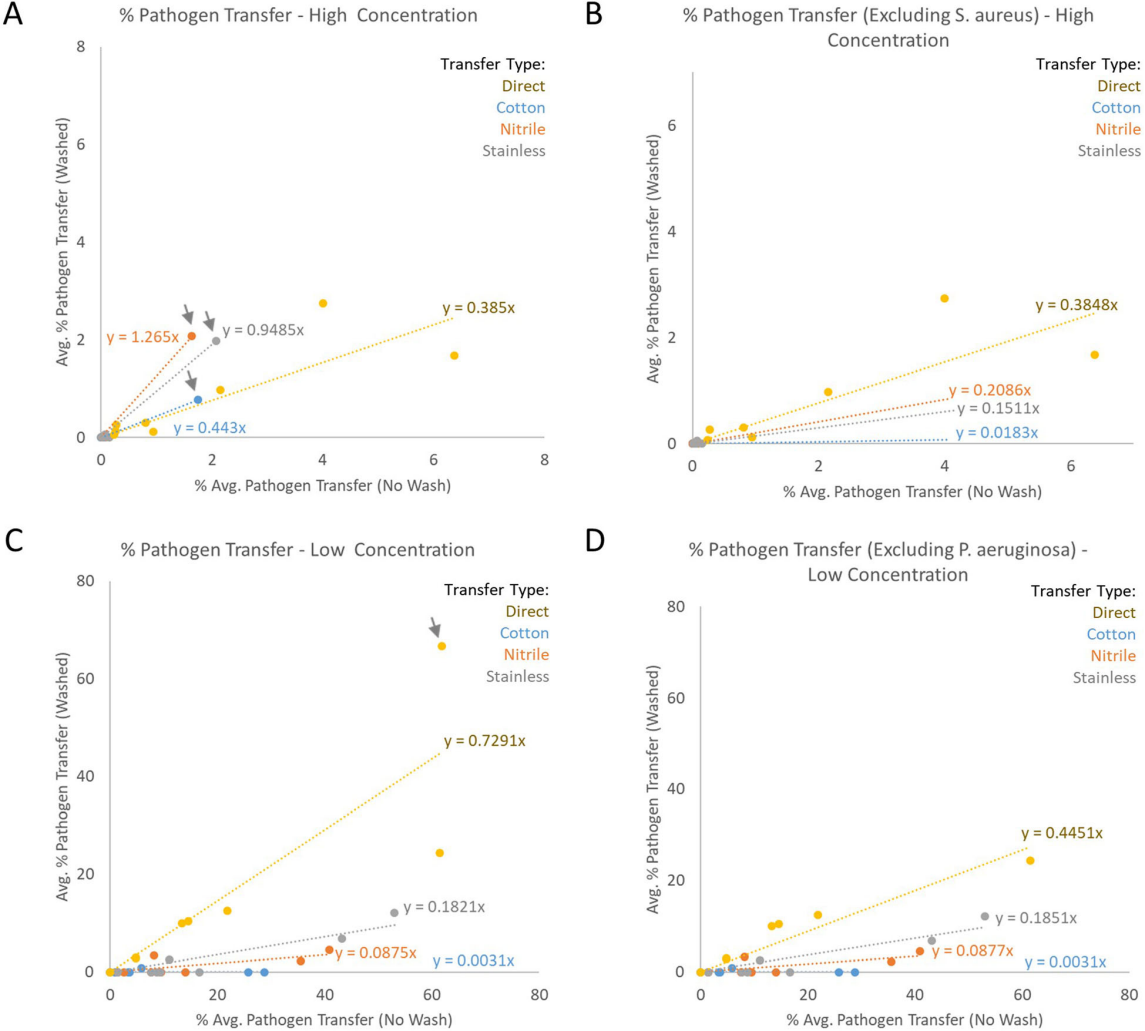
Percent pathogen transfer from initial to secondary coupon at high and low pathogen concentration. Individual transfer scenarios (direct vs. indirect) are shown. The percent transferred when no wash or decontamination step was included is represented on the X-axis, with the percent transferred following a wash or decontamination step represented on the Y-axis. Values for individual pathogens were averaged (*n* = 3) prior to plotting. Linear regression lines were plotted with the slope of each line indicated. **a** Percent pathogen transfer at high initial pathogen concentration. *S. aureus* outlier results are indicated with gray arrows. B) Percent pathogen transfer at high initial pathogen concentration excluding *S. aureus*. **b** Percent pathogen transfer at high initial pathogen concentration excluding S. aureus. **c** Percent pathogen transfer at low initial pathogen concentration. *P. aeruginosa* outlier results are indicated with a gray arrow. D) Percent pathogen transfer at low initial pathogen concentration excluding *P. aeruginosa*

Figure 7a suggests that, in high inoculum samples, the success of decontamination was limited, especially on nitrile and stainless steel surfaces. Essentially equivalent amounts of pathogen were transferred across nitrile or stainless steel fomites regardless of the use of disinfectant wipes. Laundering the cotton surface or hand washing the direct contact samples decreased overall pathogen transmission rates approximately 60%. However, the indirect transfer results were heavily skewed by a single pathogen, *S. aureus*. Removing the *S. aureus* outlier (Fig. 7b) reveals that the surface decontamination methods were reasonably effective for each of the other ESKAPE+C pathogens, reducing overall pathogen transmission by at least 80% in each case and nearly eliminating pathogen transmission across the laundered cotton fomite. These results align with previous findings regarding *S. aureus* and decontamination approaches on skin and related surface types [12, 15, 16]. The removal of *S. aureus* did not alter the overall direct transfer rates, suggesting the higher rate of *S. aureus* transfer in indirect scenarios arises from resistance to the fomite decontamination methods used.

Figure 7c demonstrates similar results for the low concentration pathogen samples. At low pathogen inoculum levels, fomite decontamination was largely effective, reducing pathogen transfer rates between 89 and 99%. In contrast to the high concentration results, *S. aureus* was not an outlier at low inoculum levels. However, the transfer rate of *P. aeruginosa* was an outlier at the low inoculum level specifically for direct transfer, demonstrating nearly equivalent transfer rates in washed versus unwashed scenarios. Excluding *P. aeruginosa* from the analysis (Fig. 7d) decreased overall pathogen transfer rates for low inoculum direct transfer to levels approximating the high inoculum levels (compare yellow line between Fig. 7a and d). *P. aeruginosa* transfer is commonly linked to hand hygiene and these results may be indicative of bacterial persistence on the hand following washing [17–19]. Exclusion of *P. aeruginosa* did not substantially affect the overall transfer rates observed in the indirect contact scenarios.

## Discussion

The prevalence of healthcare-acquired infections (HAI) and rising levels of antimicrobial resistance places a significant economic burden on modern healthcare systems [1, 20]. Epidemiological studies are commonly used to model or track pathogen transmission within the healthcare environment [21], but these studies are often limited by the amount of available data. Relevant synthetic systems, such as the one we describe, are critical to model and understand the transmission of pathogens in clinical settings because they do not require the involvement of human subjects. Such systems permit the generation of precise, quantitative data regarding pathogen transmission rates without risking the health of human subjects. The degree to which synthetic alternatives mimic the properties of human skin remains an open question worthy of further research.

The use of surrogate skin to understand pathogen transfer is not new, but such approaches may not be broadly relevant to the clinical setting due to the contrived nature of the sample [12, 22]. By producing and evaluating a method that incorporates a background microbial flora in addition to specific pathogens of interest, we have provided an additional tool in the arsenal of epidemiologists to study pathogen transmission within the healthcare setting in a way that could potentially be used to mimic the biology of the human hand. Additional work is required to establish the degree to which the VITRO-SKIN substrate mimics the human hand. While our method incorporates a background commensal skin microbial community to better model that of a human hand, adhesion of this community to the artificial surface may be different than adhesion to human skin. The properties and pressures of microbial growth on human skin over time likely cannot be fully recapitulated by the artificial surface. Future studies are needed to establish whether the artificial surface can support sustained microbial growth. If so, it may be possible to more accurately model the sudden introduction of pathogen (e.g., *C. difficile*) onto a hand with an established skin microbiome community.

By incorporating each of the ESKAPE+C pathogens into this testing, it was possible to compare relative transfer rates in both direct and indirect scenarios. *S. aureus* demonstrated a consistently higher transfer rate in the indirect contact scenarios, including persistence during decontamination or washing. It is unclear if this finding arises from surface properties of *S. aureus* that promote transfer or more robust fitness in comparison to the other pathogens used in the study resulting in greater viability during culture [23, 24]. *E. faecium* transferred at a higher rate in the low inoculum samples, with the total transferred CFU values nearly identical in direct contact scenarios despite the different input amounts (Fig. 4). Additional study is necessary to determine whether these findings are related to the biology of *E. faecium* or represent an artifact of the transfer and recovery method used in this study.

Several patterns emerged when evaluating pathogen transfer and the relative effectiveness of decontamination across transfer scenarios. As expected based on previous studies, *S. aureus* proved resistant to simulated decontamination on fomite surfaces, especially when present at high initial concentrations on nitrile and cotton (Fig. 6b and Fig. 7) [12, 15, 16]. *P. aeruginosa* persisted in a robust manner following washing or decontamination across direct and indirect transfer (Fig. 6b). This pattern emerged in a particularly clear fashion when comparing transfer rates of all pathogens for direct transfer at low concentration where *P. aeruginosa* appeared as an outlier in the overall trend of decontamination rates (Fig. 7c and d). It is not clear whether these results reflect underlying biological characteristics of these specific pathogens or could be due to artifacts arising from the specific decontamination procedures or artificial skin system used herein. Previous studies suggest that the properties of VITRO-SKIN may not perfectly align with those of human skin as it relates to the transfer of viruses [22]. It remains to be determined if the patterns observed herein are based on the intrinsic cellular properties of the pathogens and are not due to artifacts of VITRO-SKIN, the decontamination approaches, or insufficient statistical power for the study. While these variables must be taken into account in future studies, the artificial skin system we describe appears to show promise for the discovery of molecular mechanisms by which these pathogens persist and transfer in healthcare settings.

Pathogen transfer rates were significantly higher in low inoculum samples than in high. While it is unclear what factor or factors caused this difference, it is possible that higher loadings led to the formation of clumps of bacteria instead of individual cells or thin layers. This may have prevented much of the bacteria present on the coupon from contacting the secondary coupon, limiting the amount transferred. Future efforts will be required to characterize the deposition of different amounts of bacteria on the surface of VITRO-SKIN and to devise methods to achieve even distribution of bacteria across the surface in a way that recapitulates a human skin sample.

While this study investigated various decontamination methods relevant to pathogen transfer in healthcare settings, this study was not intended to serve as an evaluation of these methods or to provide recommendations on their effectiveness. Instead, a limited number of relevant decontamination methods were included to show the value of this model in future studies and to grapple with the influence of the inclusion of a background flora on transfer and decontamination rates. The limitations imposed by our simulated handwashing method were apparent. By not duplicating the force of water pressure on the artificial skin surface, a significant amount of pathogen transmission still occurred in the “washed” samples; generally, only a 30–60% reduction in transmission when compared to the unwashed controls. Hand washing procedures specified by the CDC instruct individuals to wash their hands with running water, where the force of the water has been shown to reduce bacterial loads. Without the force of running water in our scenario, more bacteria can remain on the hands even with the friction applied while rubbing and soaking in water afterwards [14, 25, 26]. Additionally, as described in the Materials and Methods, the VITRO-SKIN was not dried completely after the simulated-hand washing. This may have aided in bacterial transfer, similar to how residual moisture on hands has the potential to transfer bacterial loads more than dried hands [25, 26]. The low contact time associated with the application of antimicrobial soap may have also played a role in the relative low success of hand washing observed in this study. Ultimately, uncovering challenges with the decontamination methods utilized in this study highlights the utility of the VITRO-SKIN system for the evaluation of factors relating to the effectiveness of handwashing and decontamination in future studies.

## Conclusions

Here, we describe the development of an in vitro method that utilizes a synthetic skin surrogate in addition to a defined, customizable microbial community mimicking the human skin flora. This approach allows various pathogen transmission pathways to be quantified, including skin-to-skin and skin-to fomite-to skin contact scenarios, in a manner that does not involve human subject testing. Our results illustrate the value of simulating a holistic microbial community for transfer studies by elucidating differences in different pathogen transmission rates and resistance to common decontamination practices. Indeed, multiple differences in transmission rates between individual ESKAPE+C pathogens were identified suggesting areas for future research focus. Ultimately, we believe this method will contribute to improvements in pathogen transmission modeling in healthcare settings. Future work should be performed to establish the concordance between pathogen transfer on hands and synthetic matrices, and to verify that the relative transmissibility of individual pathogens described herein is extensible to the clinical environment.

## Methods

### Bacterial culturing

Microorganisms used for this effort were sourced from ATCC or the CDC & FDA Antibiotic Resistance Isolate Bank (Table 1) [11].

All microorganisms were propagated according to CDC and ATCC guidelines (Table 2). An Environmental Protection Agency (EPA) Standard Operating Procedure (SOP) was used as a guideline for the preparation of *C. difficile* endospores [27]. Greater than 90% of the bacterial preparation were spores. All media and media supplements were acquired from Teknova and anaerobic equipment from BD.

**Table 2:** Bacterial growth conditions

Microorganism	Growth Media	Growth Conditions
*B. linens* ATCC 9172	Tryptic Soy Broth	Aerobic, 30 °C
*C. matruchotii* ATCC 14265	Brain Heart Infusion Broth with 2% yeast extract	Aerobic, 36 °C
*P. acnes* ATCC 11827	Tryptic Soy Broth with 5% Sheep’s Blood	Anaerobic, 36 °C
*E. coli* ATCC 9637	Tryptic Soy Broth	Aerobic, 36 °C
*L. gasseri* ATCC 33323	Lactobacilli MRS Broth	Aerobic, 36 °C
*M. luteus* ATCC 4698	Tryptic Soy Broth	Aerobic, 30 °C
*S. epidermidis* ATCC 12228	Tryptic Soy Broth	Aerobic, 36 °C
*S. pyogenes* ATCC 19615	Tryptic Soy Broth with 5% Sheep’s Blood	5% CO_2_, 36 °C
*A. baummannii* AR Bank 275	Tryptic Soy Broth	Aerobic, 36 °C
*E. faecium* AR Bank 579	Tryptic Soy Broth	Aerobic, 36 °C
*K. pneumoniae* AR Bank 139	Tryptic Soy Broth	Aerobic, 36 °C
*E. aerogenes* AR Bank 161	Tryptic Soy Broth	Aerobic, 36 °C
*S. aureus* AR Bank 219	Tryptic Soy Broth	Aerobic, 36 °C
*C. difficile* (endospores) ATCC 43598	Endospore Preparation	Anerobic, 36 °C
*E. cloacae* AR Bank 365	Tryptic Soy Broth	Aerobic, 36 °C
*P. aeruginosa* AR Bank 230	Tryptic Soy Broth	Aerobic, 36 °C

### Determination of bacterial count

To facilitate rapid viability screening using culture methods, three separate mixtures were created for testing (Table 3). The viability of each ESKAPE+C pathogen was assessed on three selective medias: mannitol salt agar (MSA), eosin methylene blue (EMB) agar, and *C. difficile* agar, with the indicated antibiotics included (Acros Organics for gentamicin and Alfa Aesar for ampicillin) (See Table 3). Plates were supplemented with antibiotics and allowed to dry overnight prior to testing. Bacteria were enumerated by serial dilution in PBS (Teknova) and subsequent plate count following incubation for 72–96 h at 36 °C. *C. difficile* growth occurred under anaerobic conditions.

**Table 3:** Bacterial mixtures for testing

Mixture	Selective Agar	Bacteria
1	MSA and EMB with 16 μg/mL of Ampicillin	Background (See Table 1)
		*A. baumannii*
		*E. faecium*
		*K. pneumoniae*
2	MSA with 24 μg/mL Gentamicin EMB with 8 μg/mL of Gentamicin	Background (See Table 1)
		*E. aerogenes*
		*S. aureus*
3	MSA and EMB with 8 μg/mL of Gentamicin *C. difficile* Agar	Background (See Table 1)
		*C. difficile* endospores
		*E. cloacae*
		*P. aeruginosa*

### Preparation of synthetic skin and indirect testing surfaces

VITRO-SKIN N-19 (IMS Inc.) was used to simulate both skin-to-skin (i.e., direct) and skin-to fomite-to skin (i.e., indirect) transmission. All VITRO-SKIN sheets were cut into 3 × 3 cm coupons prior to hydration. VITRO-SKIN was used within 24 h of hydration. Stainless steel coupons (3 × 3 cm) (Home Depot) were cleaned with 70% isopropyl alcohol, rinsed with deionized (DI) water, and allowed to dry. After drying, surfaces were steam sterilized by autoclaving prior to testing. Nitrile gloves (VWR) and cotton surfaces were cut to 3 × 3 cm, placed in a sterile container exposed to UV light for approximately 1 h. Sterilized surfaces were used within 24 h. Cotton surfaces were cut from a new laboratory cotton/polyester blend coat. Negative controls (VITRO-SKIN coupons not inoculated with bacteria) were included to confirm that no ESKAPE+C pathogens were present on sterilized fomite surfaces.

### Preparation of VITRO-SKIN with bacterial inoculum

At the time of testing, pathogenic and background cultures were pelleted by centrifuging at 5000 x g for 10 min (excluding anaerobic microorganisms or cultures prepared in sheep’s blood) and resuspended in 2 mL of PBS. Resuspended bacteria were combined per the appropriate mix (Table 3) at the appropriate spike-in level and brought up to 5 mL using PBS. Spike in target levels were ~ 10^6^ CFU per VITRO-SKIN for “high” ~ 10^2^ CFU for “low.” Actual spike in values were measured and reported (Fig. 2). On the day of testing, one mix containing pathogens plus background and one mix containing only background microorganisms was prepared. Once the appropriate pathogen and background mixes were prepared, 0.100 mL of a bacterial mix was added per hydrated VITRO-SKIN coupons and spread evenly using a sterile spreader, avoiding edges. The inoculated coupons were dried at 36 °C for 45 min or until visibly dried.

### Simulation of direct contact scenario

For the no-wash direct contact scenario, a primary VITRO-SKIN coupon containing a pathogen mix was touched to a secondary VITRO-SKIN coupon containing only background microbiota by evenly applying pressure for 10 s using sterile forceps. The secondary VITRO-SKIN coupon was then added to 10 mL PBS and vortexed for 3 min to release the bacteria. Recovered bacteria was then appropriately diluted and plated on selective media. For simulated hand washing, a procedure was adapted from Arinder et al. [12] To a pathogen-inoculated VITRO-SKIN coupon, 0.100 mL of hospital-grade hand soap (Thermo Scientific SoftCide Hand Soap) was applied to the bacterial-laden surface. A second pathogen-inoculated VITRO-SKIN coupon was pressed evenly onto the first, allowing the soap to spread evenly between both coupons. The coupons were rubbed against each other 20 times to achieve the same amount of foam as normal hand washing. After washing, the secondary VITRO-SKIN was rinsed sequentially using two separate 10 mL conical tubes containing sterile PBS. Following the second rinse, the coupon was rinsed in 10 mL of Dey-Engley (D/E) broth. After the D/E broth rinse, the washed VITRO-SKIN was touched to a new VITRO-SKIN coupon that contained only background microbes by evenly applying pressure for 10 s using sterile forceps. The new VITRO-SKIN with the transferred bacteria was added to 10 mL of PBS, vortexed for three minutes, and appropriately diluted and plated on selective media.

### Simulation of indirect contact scenario

Indirect contact events were performed in a similar manner as direct contact events (Fig. 1b). Dried and inoculated VITRO-SKIN samples were prepared and touched to one of the three different surface types (i.e., nitrile gloves, cotton cloth to represent a patient bedsheet, and stainless steel to represent medical equipment). This testing occurred with and without a decontamination step on the surface following the initial transfer across two pathogen spike-in levels. The cleaning procedure for stainless steel and gloves was performed with a fresh, broad spectrum hospital disinfectant wipe per manufacturer instructions (Metrex Caviwipes Bleach®). The cloth surfaces were cleaned with a simulated hot-water cleaning to replicate how textiles are cleaned in a hospital washing machine. Afterward, a secondary coupon containing only the background microbiome was touched to the cleaned or non-cleaned surface. The simulated hot water cloth cleaning procedure was adapted from “A Sanitary Study of Commercial Laundry Practices,” recommended by the CDC [28]. All replicates of soiled cotton coupons were placed in a sterile beaker with a sterile stir bar containing 200 mL of sterile water to ensure all cloth replicates were submerged and were rinsed for 5 min at 43 °C. After first rinse, the water was dispensed into a waste container to be autoclaved. Sterile water (200 mL; 0.027 μL/mL stock detergent (All® Free & Clear) was added to a new, sterile beaker to ensure all cotton replicates were submerged and allowed to stir to provide agitation for 10 min at 52 °C. Afterward, 2% bleach was added to the mixture and was allowed to stir to provide agitation for 15 min at 78 °C. The water was then removed and placed in a waste container for autoclaving. Sterile water (200 mL) was added to a new, sterile beaker to ensure all cotton replicates were submerged and was allowed to stir for 3 min at 78 °C. The water was removed appropriately and placed in a waste container for autoclaving. The wash step for 3 min at 78 °C was repeated twice. After the final rinse, each cotton replicate was placed in an appropriate sterile container and placed in an oven at 70 °C for 30 min or until visibly dried. Once dried, each replicate was added to 10 mL PBS, vortexed for 3 min, and enumerated per standard spread plating technique.

### Culture analysis

For each pathogen mixture and simulated contact scenario, the following controls were harvested and plated: one primary inoculated VITRO-SKIN as the pre-transfer control; one clean VITRO-SKIN (i.e., no background skin microbiome or ESKAPE+C pathogens) as the negative control; one clean or sterile surface (i.e. fomite) per surface type as a negative control. One sterile agar plate per agar type was incubated alongside test plates to ensure plate sterility. Each pathogenic microorganism per mixture type per testing simulation was struck for isolation to ensure there was no contamination. A volume of 0.100 mL of each dilution buffer, harvesting buffer, etc. was plated onto sterile TSA to ensure no contamination of each respective buffer at the time of testing.

Following the transmission event simulations, each replicate was aseptically placed into separate conicals containing 10 mL of PBS and vortexed at max speed for at least 3 min. Afterward, individual 1 mL aliquots (*n* = 2) were plated directly onto the appropriate selective media and incubated at the appropriate temperatures. Colonies were counted manually to quantify the number of CFUs.

### Viability and experimental calculations

Equations 1 and 2 below were used for culture-dependent method analysis. Equation 1 was used to determine the number of viable bacteria of a given strain on the surface of a VITRO-SKIN carrier following elution in 10 mL of buffer. Eq. 2 was used to generate the average CFU per carrier across multiple experimental replicates.

1 $$ CFU\  per\  Carrier=\left[\left( Dilution\ Plate\#1+ Dilution\ Plate\#2\right)/2\right]\ x\  DF\ x\  Elution\ Volume $$

Equation 1 CFU per Carrier

2 $$ \frac{\begin{array}{c}\Big( CFU\  per\  Carrier\ Replicate\#1+ CFU\  per\  Carrier\ Replicate\#2+ CFU\  per\  Carrier\ Replicate\#3+\\ {} CFU\  per\  Carrier\ Replicate\#N\Big)\end{array}}{Total\ Replicate\ Number} $$

Equation 2 Average CFU per Carrier

### Statistical analysis

Statistical analysis of Log difference between the control VITRO-SKIN and the resulting VITRO-SKIN from transfer of the eight ESCKAPE+C pathogens were performed by ANOVA using multiple factors as described. The analysis was performed using the ‘aov’ function in ‘R’ statistical package through ‘Rstudio’ [29]. The ANOVA was followed by a Tukey Honest Significant Difference Test utilizing the ‘TukeyHSD’ function. The detection limit for CFU counts were used for values below that limit.

## Supplementary information

**Additional file 1.**

## Data Availability

All data generated or analyzed during this study are included in this published article and its supplementary information files.

## Abbreviations

HAI: Healthcare-acquired infections
ESKAPE: *Enterococcus faecium, Staphylococcus aureus, Klebsiella pneumoniae, Acinetobacter baumannii, Pseudomonas aeruginosa,* and *Enterobacter* species
ESKAPE+C: *Enterococcus faecium, Staphylococcus aureus, Klebsiella pneumoniae, Acinetobacter baumannii, Pseudomonas aeruginosa, Enterobacter* species and *Clostridioides difficile*
CDC: Centers for disease control and prevention
FDA: Food and drug administration
AR: Antibiotic resistance
CFU: Colony forming units
ANOVA: Analysis of variance
PBS: Phosphate buffered saline
EPA: Environmental protection agency
SOP: Standard operating procedure
MSA: Mannitol salt agar
EMB: Eosin methylene blue
D/E: Dey-Engley
DI: Deionized
